# Clinical and laboratory characteristics of hemophagocytic lymphohistiocytosis induced by *Leishmania infantum* infection

**DOI:** 10.1371/journal.pntd.0009944

**Published:** 2021-11-04

**Authors:** Qi Shi, Minjun Huang, Xiaoli Li, Xiaoyan Zheng, Fei Wang, Yang Zou, Lei Wang, Jidong Jia

**Affiliations:** 1 Beijing Institute of Tropical Medicine, Beijing Friendship Hospital, Capital Medical University, Beijing, P.R. China; 2 Liver Research Center, Beijing Friendship Hospital, Capital Medical University, Beijing, P.R. China; 3 Beijing Key Laboratory for Research on Prevention and Treatment of Tropical Diseases, Beijing, P.R. China; 4 Beijing Key Laboratory of Translational Medicine on Liver Cirrhosis, Beijing, P.R. China; 5 National Clinical Research Center of Digestive Diseases, Beijing, P.R. China; University of Texas Medical Branch, UNITED STATES

## Abstract

**Background:**

Visceral leishmaniasis (VL) could progress to secondary hemophagocytic lymphohistiocytosis (HLH), which is a rare but life-threatening condition with poor prognosis. So far, the clinical and laboratory characteristics of VL associated HLH have not been well elucidated.

**Method and findings:**

In this study, we retrospectively analyzed the clinical and laboratory profiles between 17 patients with VL associated HLH and 27 patients with VL alone admitted at the Beijing Friendship Hospital, Capital Medical University from May 2016 to March 2021. In addition to the identification of *Leishmania* infection, hemophagocytosis was identified in bone marrow in the most cases of VL associated HLH (15/17). The patients with VL associated HLH had higher chances of bleeding, hepatomegaly, thrombocytopenia, hypertriglyceridemia, hyperferritinemia, hypofibrinogenemia, elevated secretion of soluble IL-2 receptor or lower NK cell activity compared to patients with VL only. Furthermore, patients with VL associated HLH had higher inflammation status associated with higher levels of Th1 (TNF-α, IFN-γ, IL-1beta, IL-6, IL-8, IL-12p70), Th2 (IL-4) and Th17 cytokines (IL-17, IL-23) in the peripheral blood, and higher parasite load (qPCR and parasite culture). All 27 VL cases were totally recovered after being treated with Sodium Stibogluconate, five of the 17 patients with VL associated HLH died even after timely treatment with anti-parasite and immunosuppressive chemotherapy.

**Conclusion:**

Without appropriate treatment, visceral leishmaniosis could develop to secondary HLH. The parasite culturing and qPCR detection of bone marrow samples facilitates the diagnosis of VL associated HLH in addition to other findings of HLH. Prompt treatment with anti-*Leishmania* and immunosuppressive chemotherapy is critical to reduce the mortality of VL associated HLH.

## Author summary

Hemophagocytic lymphohistiocytosis (HLH) is a rare but life-threatening syndrome characterized by pro-inflammatory cytokine secretion, hyperinflammatory and multiple organ damages. Visceral leishmaniasis (VL) is a well-known cause of infection associated HLH and result in fatal consequence. However, it is not well characterized for the clinical and laboratory features of the visceral leishmaniasis associated HLH. In this study, we presented that, compared to the VL alone, patients with VL associated HLH had higher chances of bleeding, hepatomegaly, thrombocytopenia, hypertriglyceridemia, hyperferritinemia, hypofibrinogenemia, elevated secretion of soluble IL-2 receptor or lower NK cell activity. Moreover, patients with VL associated HLH also had higher levels of Th1, Th2 and Th17 cytokines in the sera and had higher parasite load in the bone marrow specimen. More cases with VL associated HLH had hepatosplenomegaly with iron overload in the magnetic resonance imaging. In the therapeutic strategy, besides the anti-*Leishmania* treatment, anti-inflammatory therapy to reduce cytokine storm and excessive immune responses facilitated the remission in the VL associated HLH cases.

## Introduction

Visceral leishmaniasis (VL), also known as Kala-azar, is a zoonotic disease caused by the infections of *Leishmania donovani* or *L*. *infantum*, listed as one of the most serious neglected tropical diseases by the World Health Organization (WHO) [1]. More than 350 million people are at risk of *Leishmania* infection globally with approximate 50,000 to 90,000 new cases of VL occurred worldwide annually, of which 26,000 to 65,000 died each year [2]. In 2018, more than 95% of new cases reported in India, Brazil, Ethiopia, Kenya, Somalia, Sudan, and South Sudan [3]. However, the number of infected people are seriously under-estimated with only 20–45% reported to WHO [1]. Without adequate and timely treatment, over 95% VL patients would die within 2 years due to the serious complications including hemophagocytic lymphohistiocytosis (HLH) or secondary infections [4].

Hemophagocytic lymphohistiocytosis is a life-threatening syndrome characterized by uncontrolled and dysfunctional activation of T lymphocytes, natural killer (NK) cells, and macrophages by continuous antigen stimulation, resulting in hypercytokinemia (cytokine storm) and immune-mediated injury of multiple organ systems [5,6]. The primary HLH is a hereditary immune disorder that typically occurs in infants caused by the mutations in genes activated in cytotoxic T-lymphocyte and natural killer-lymphocyte. The secondary HLH develops as a complication in various settings including autoimmune disorders, various infections, and malignancies [7].

*Leishmania* infection is a well-known cause of infection associated HLH [4,8–11]. It was reported that about 25.77% of patients with adult hemophagocytic syndrome were induced by *Leishmania* infection [12]. As many as 41.7% of children with visceral leishmaniasis developed HLH if lack of timely treatment and the mortality rate was as high as 100% [4,9]. The clinical features of VL overlap with that of HLH that challenges the differential diagnosis of VL and VL associated HLH especially in endemic areas of VL. Consequently, *Leishmania* infection might be neglected and the intense immunosuppression targeting HLH might be administrated without specific antimicrobial therapy for Leishmaniasis that may lead to the disastrous consequences.

Up to now, the characteristics of VL associated HLH has not been well described. Therefore, in this study, we analyzed the clinical and laboratory characteristics of HLH induced by *L*. *infantum* infection, with the aim to facilitate the early diagnosis and timely treatment, thereby improving the prognosis of patients with VL associated HLH.

## Patients and methods

### Ethics statement

This project was approved by the Ethics Committee of Beijing Friendship Hospital, Capital Medical University for Human Research (Beijing, China) with approval number of 2020-P2-005-01. A written informed consent was obtained from each participant involved in the study to collect general information, biological samples, radiological and pathological images for research purpose. The identity of each participant was protected and will never be released.

### Study design, diagnostic procedures and inclusion criteria

This retrospective analysis was conducted on patients diagnosed with VL with or without HLH who were admitted to the Beijing Friendship Hospital, Capital Medical University between May 2016 and March 2021.

The VL diagnosis was made according to the recommendations of the Ministry of Health Guideline including the parasite identification, serological positive for rK39 or PCR positive for *Leishmania* DNA [13]. Parasitological identification was based on the detection of amastigotes in the bone marrow smears stained with Giemsa or the isolation of promastigotes from the culture of bone marrow sample in Novy-MacNeal-Nicolle (NNN) media. Immunological assays were performed to test IgG antibody against rK39 antigen (kalazar Rapid Test, InBios, Seattle, WA, USA) in sera. A regular PCR or quantitative real-time PCR (qPCR) were used to amplify kinetoplast minicircle DNA (mkDNA) of *Leishmania* in bone marrow specimen using methods previously described [14]. Briefly, DNA was extracted from bone marrow using a TIANGEN DNA extraction kit (TIANGEN, DP705, Beijing, CHN) and primers based on the mkDNA conserved region were used for regular PCR or qPCR detection [14]. The diagnosis of VL was made if at least one of the three laboratory tests were positive (parasite identification, serological positive for rK39 or PCR positive for *Leishmania* DNA). The VL patient inclusion criteria in this study included positive for parasite detections as above, in addition to the symptoms of infection (fever, fatigue, etc) and epidemiological correlation (from endemic area and having history of sand fly bites).

The inclusion criteria for VL associated HLH patients should meet five or more out of eight criteria set in diagnostic guidelines for HLH (HLH-2004 criteria) (**Table 1**) [15] in addition to the identification of *Leishmania* parasite (microscopy, antibody or PCR).

**Table 1:** Diagnostic guidelines for HLH (HLH-2004 criteria) [15].

Five criteria in 1991 guideline (must be fulfilled for all HLH patients) 1. Fever 2. Splenomegaly 3. Cytopenias affecting at least two of three lineages in the peripheral blood 4. Hypertriglyceridemia and/or hypofibrinogenemia 5. Hemophagocytosis in bone marrow, spleen, or lymph nodes Three new criteria added in 2004 guideline (optional) 6. Low or absent NK-cell activity 7. Hyperferritinemia 8. High levels of sIL-2 receptor (soluble CD25)

### Cytokine measurement by Luminex assay

Sera were collected from VL patients (N = 27) and VL associated HLH patients (n = 17) for measuring Th1 cytokines (TNF-α, IFN-γ, IL-1beta, IL-6, IL-8, IL-12p70), Th2 cytokines (IL-4, IL-10) and Th17 cytokines (IL-17, IL-23) using Human Magnetic Luminex Assay (R&D Systems, Minneapolis, MN, USA). Samples were run on a Bio-Plex Magpix multiplex reader according to manufacturer’s recommendations (Luminex, Austin, TX). Raw Luminex data were analyzed using the Bio-Plex Manager 6.0 software and plotted in GraphPad Prism 8.0. Signal background in blank media was subtracted from re-stimulated samples.

### Treatment of VL with or without HLH and follow-up

All *Leishmania* infected patients were intramuscularly treated with anti-*Leishmania* Sodium Stibogluconate (SSG), the first line therapeutic drug recommended by Ministry of Health of China [13]. Based on the treatment guideline, a 6-day treatment course with 60 mg/kg for adults and 120 mg/kg for children in total amount (or 10 mg/kg/day for adults and 20 mg/kg/day for children for a consecutive 6 days) was conventionally used to treat patients with VL [13]. If the symptoms persisted and the parasite detection remained positive, another treatment course was given until parasites were totally eliminated.

The patients with VL associated HLH were additionally received chemotherapy with methylprednisolone, cyclosporine A and etoposide sequentially according to the therapeutic guideline HLH-2004 [15].

Each patient in this study was participated in the follow-up program for at least six months with four weeks interval. The follow-up information included symptoms, blood test for complete blood count (CBC) and biochemical profiles and ultrasound examination for liver and spleen.

### Data collection

The data were collected and input in the database, including clinical data, laboratory examinations (blood CBC, biochemical test). imaging characteristics and etiological information of *Leishmania*. Patients with incomplete data, complicated with malignant tumor, autoimmune diseases or with other infections were excluded from this analysis.

### Statistical analysis

Statistical analysis was carried out using the SPSS software version 21.0 and visualized on GraphPad Prism version 8.0. Continuous variables were described as means ± standard deviations. Categorical variables were summarized with absolute and relative frequencies. To compare percentages, we used Chi square test. Means were compared using Student t-test. The Mann-Whitney *U*-test was used to statistically compare between groups. *P* <0.05 was considered as statistical significance.

## Results

### Demographic features and clinical manifestations

**Fig 1:**
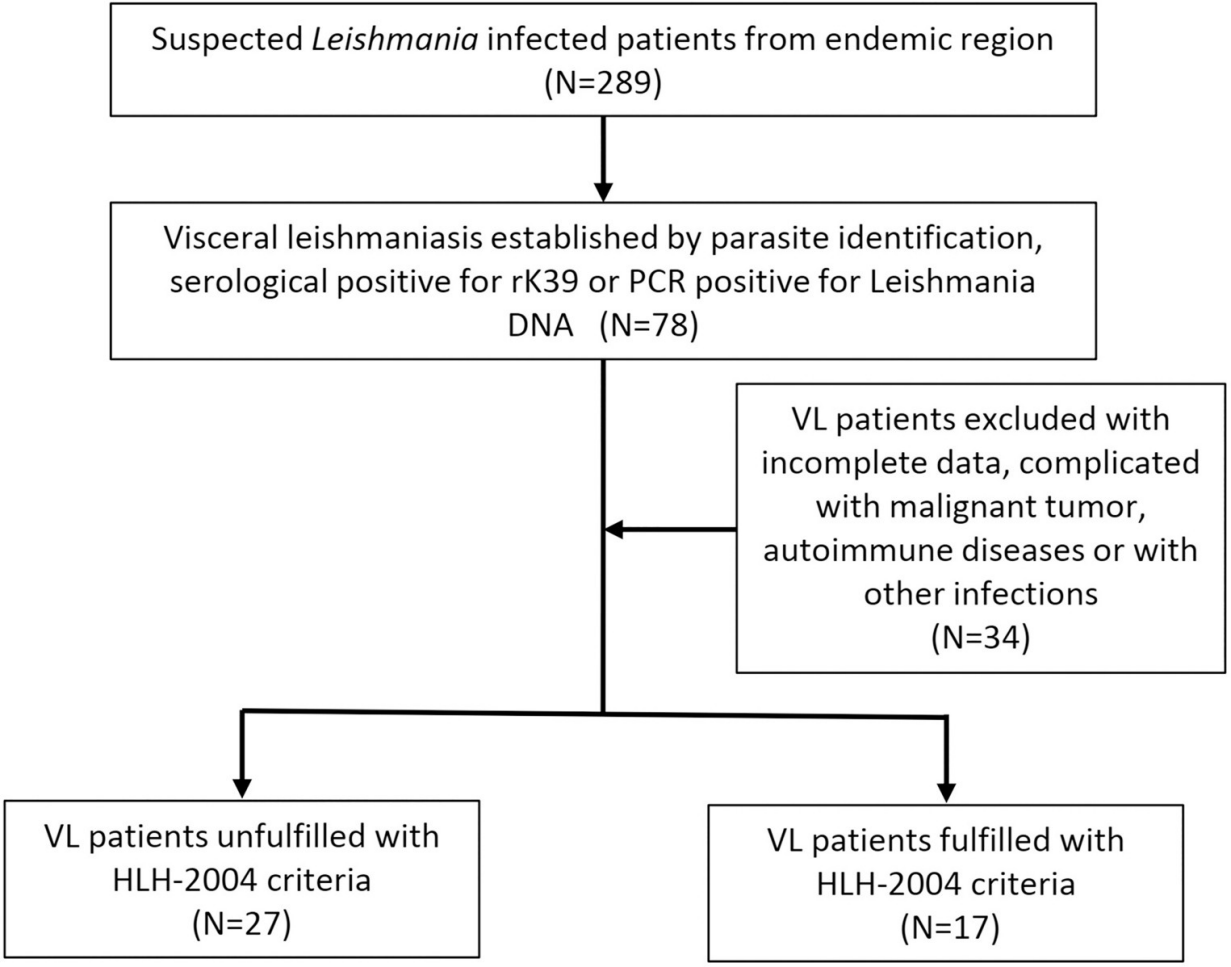
Flow chart for VL and VL associated HLH selection.

A total of 289 cases with suspected VL from endemic regions of China were admitted from May 2016 and March 2021 in our institute. Each patient received *Leishmania* diagnostic test and 78 cases were diagnosed with VL. Among them, 34 cases were excluded due to confounding factors such as incomplete data or other physical disorders. Thus, the 44 VL cases were confirmed and classified as with or without HLH based on the HLH-2004 criteria. Finally, 27 cases with VL alone and 17 cases with VL associated HLH were included in this study (**Fig 1**).

The criteria for diagnosis of HLH are listed in **Table 1**. Patients with VL met five or more out of eight criteria set in this standard were diagnosed as VL-associated HLH in this study. Total 17 VL-associated HLH patients were identified based on the diagnostic criteria of HLH-2014, 12 of them met seven out of eight criteria, one patient met six and the rest four met the minimal five criteria. On the other hand, in the 27 VL alone patients, no case met the minimum five criteria of HLH.

The comparison of major demographic features and the most common symptoms and manifestations between VL and VL associated HLH is shown in **Table 2**. Basically, most of VL and VL associated HLH cases happened in patients with age less than 5 years old or higher than 40 (77.78%, 70.59%), more male (62.9%, 76.4%) than female (37.1%, 23.6%). Most of cases came from Shanxi endemic area (85.2%, 70.6%). They all had history to be bitten by sand fly. The most common symptoms in VL and VL associated HLH patients were fever (100%, 100%), weakness (51.85%, 58.82%), malnutrition (77.77%, 88.23%) and splenomegaly (66.66% and 76.47%), respectively. In terms of malnutrition severity, 19 VL patients and 11 VL associated HLH patients had moderate malnutrition, whereas the rest of patients had severe malnutrition base on the GLIM criteria [16]. However, patients with VL associated HLH had higher chances to show bleeding (78.21% vs 29.62%) and hepatomegaly (52.94% vs 22.22%) compared to VL alone with significant difference (*P*<0.05).

**Table 2:** Comparison of demographic and clinical presentation of VL patients with or without HLH.

Features	VL alone (*n* = 27) %	VL associated HLH (*n* = 17) %
Age		
<5 or>40	21 (77.78)	12 (70.59)
5–40	6 (22.22)	5 (29.41)
Gender		
Male	17 (62.9)	13 (76.4)
Female	10 (37.1)	4 (23.6)
Residence		
Shanxi	23 (85.2)	12 (70.6)
Hebei	1 (3.7)	2 (11.8)
Gansu	3 (11.1)	3 (17.6)
History of sand fly bite	27 (100)	17 (100)
Clinical presentation		
Fever	27 (100)	17 (100)
Weakness	14 (51.85)	10 (58.82)
Bleeding	8 (29.62)	13 (78.21) *
Malnutrition	21 (77.77)	15 (88.23)
Splenomegaly	18 (66.66)	13 (76.47)
Hepatomegaly	6 (22.22)	9 (52.94) *
Lymphadenopathy	9 (33.33)	5 (29.41)

*indicates *P* value less than 0.05 between VL and VL associated HLH group.

### Conventional laboratory findings

The laboratory tests revealed abnormal pancytopenia in all patients with more severity of thrombocytopenia in the VL associated HLH group compared to VL patients (*P*<0.05). The blood biochemistry revealed different features in the two groups. The level of immunoglobulin G was significant higher in the VL associated HLH cases than those in VL patients, but no difference found in the albumin and TP level. Furthermore, the levels of triglycerides (TG) and low-density lipoprotein (LDL-C) were significantly higher in the sera of VL associated HLH cases than those in VL patients. The levels of ferritin and LDH were elevated in both groups, but was significant higher in VL associated HLH cases than in VL alone. In addition, fibrinogen and NK cell activity were much reduced, but sIL-2 receptor level was much higher in the VL associated HLH patients compared to VL patients (**Table 3**).

**Table 3:** Comparison of laboratory tests and prognosis between VL and VL associated HLH groups.

Parameters	Normal range	VL (*n* = 27)	VL associated HLH (*n* = 17)	*P*-value
Hematology				
Leukocytes (×10^9^/L)	3.5–9.5	3.46±2.48	2.60±1.07	0.68
Hemoglobin (g/L)	115–150	85.70±29.35	68.11±16.54	0.057
Platelet (×10^9^/L)	125–350	116.48±72.74	62.05±26.05*	0.039
Biochemistry				
TP (g/L)	65–85	85.85±18.95	88.17±8.24	0.624
ALB (g/L)	40–55	29.07±6.41	27.31±5.64	0.557
Immunoglobulin G (g/L)	7–16	38.05±23.01	53.26±9.88*	0.001
CHOL (mmol/L)	3.9–5.2	2.74±0.78	3.76±1.58	0.63
TG (mmol/L)	0.57–1.7	1.56±0.64	3.28±0.69*	0.013
LDL-C (mmol/L)	2.34–3.12	1.48±0.50	2.32±0.68*	0.002
Ferritin (μg/L)	24–336	381.62±51.32	7934.64±1654.77*	0.006
LDH (IU/L)	120–250	414.96±75.56	2209.88±413.02*	0.008
Fibrinogen (g/L)	>1.5	1.72±0.58	0.92±0.24*	0.018
NK cell activity (%)	10–15	14.2±3.24	8.27±2.64*	0.012
sIL-2 receptor (soluble CD25) (U/ml)	<6400	5124±562	33786±468*	0.003
Etiology				
Bone marrow smears (%)	Neg	77.77	82.35	0.52
Culture positive (%)	Neg	33.3	64.7*	0.013
rK39 IgG positive (%)	Neg	100	88.23	0.257
Conventional PCR (%)	Neg	100	100	
qPCR load (×10^6^/mL, copy of mkDNA)	Neg	1.4±0.6	8.2±6.48*	0.001
Prognosis				
Cured	-	27	12	-
Died	-	0	5	-

* indicated *P* value less than 0.05 between VL and VL associated HLH group.

### Cytokine measurement

**Fig 2:**
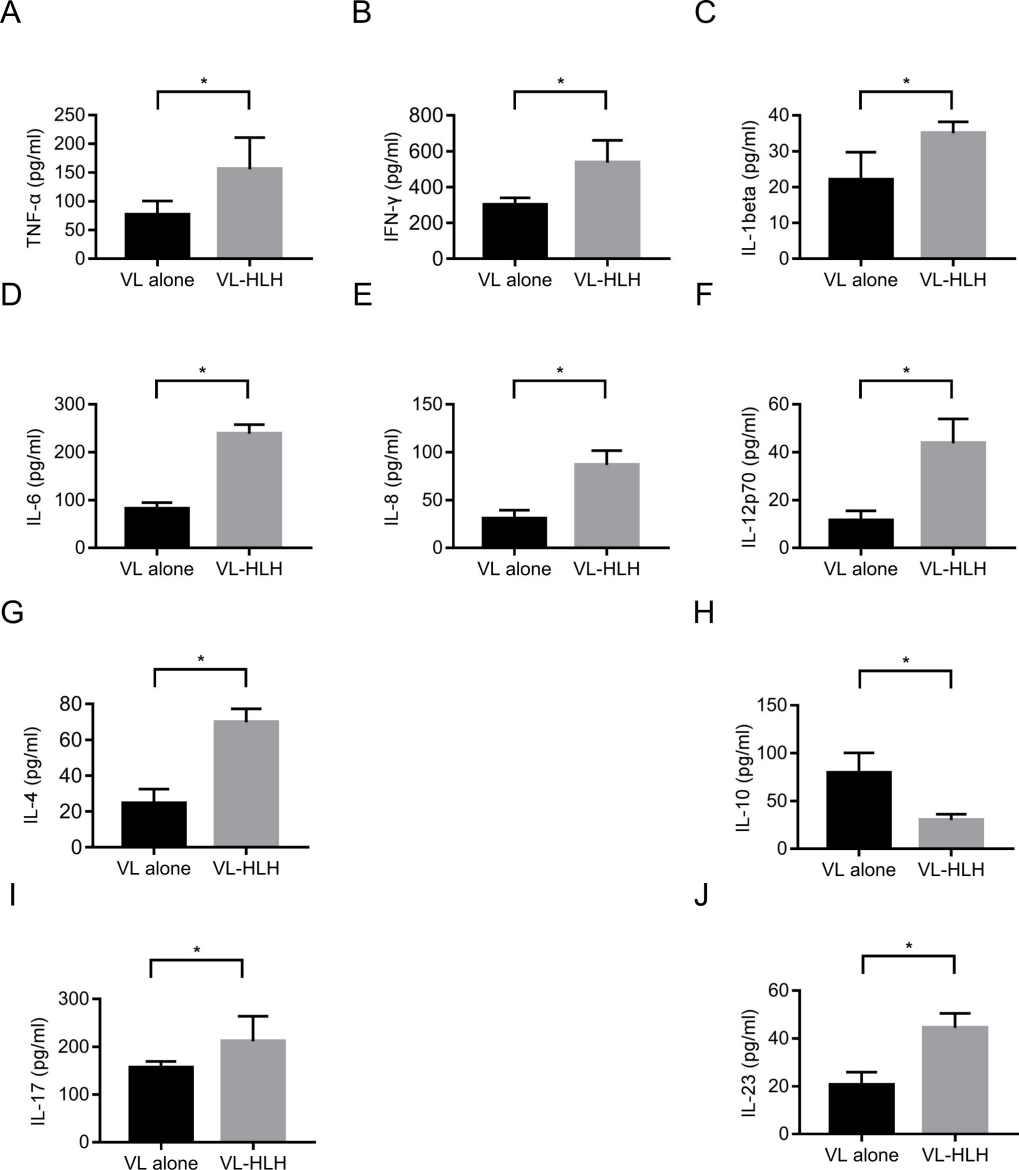
Sera cytokines measurement in the VL and VL associated HLH groups. Cytokines were measured by Luminex assay in sera of VL (n = 27) and VL associated HLH (n = 17) patients. (A) TNF-α, (B) IFN-γ, (C) IL-1beta, (D) IL-6, (E) IL-8, (F) IL-12p70, (G) IL-4, (H) IL-10, (I) IL-17 and (J) IL-23. Data are mean ± SD of three independent experiments, *P* < 0.05 between two groups.

The cytokines measurement results showed that the levels of most of Th1 cytokines (TNF-α, IFN-γ, IL-1beta, IL-6, IL-8, IL-12p70), Th2 cytokines (IL-4) and Th17 cytokine (IL-17, IL-23) were significantly higher in VL associated HLH group than those with VL only except for the regulatory cytokine IL-10 which was higher in VL group than in VL associated HLH group (**Fig 2**).

### Imaging study

**Fig 3:**
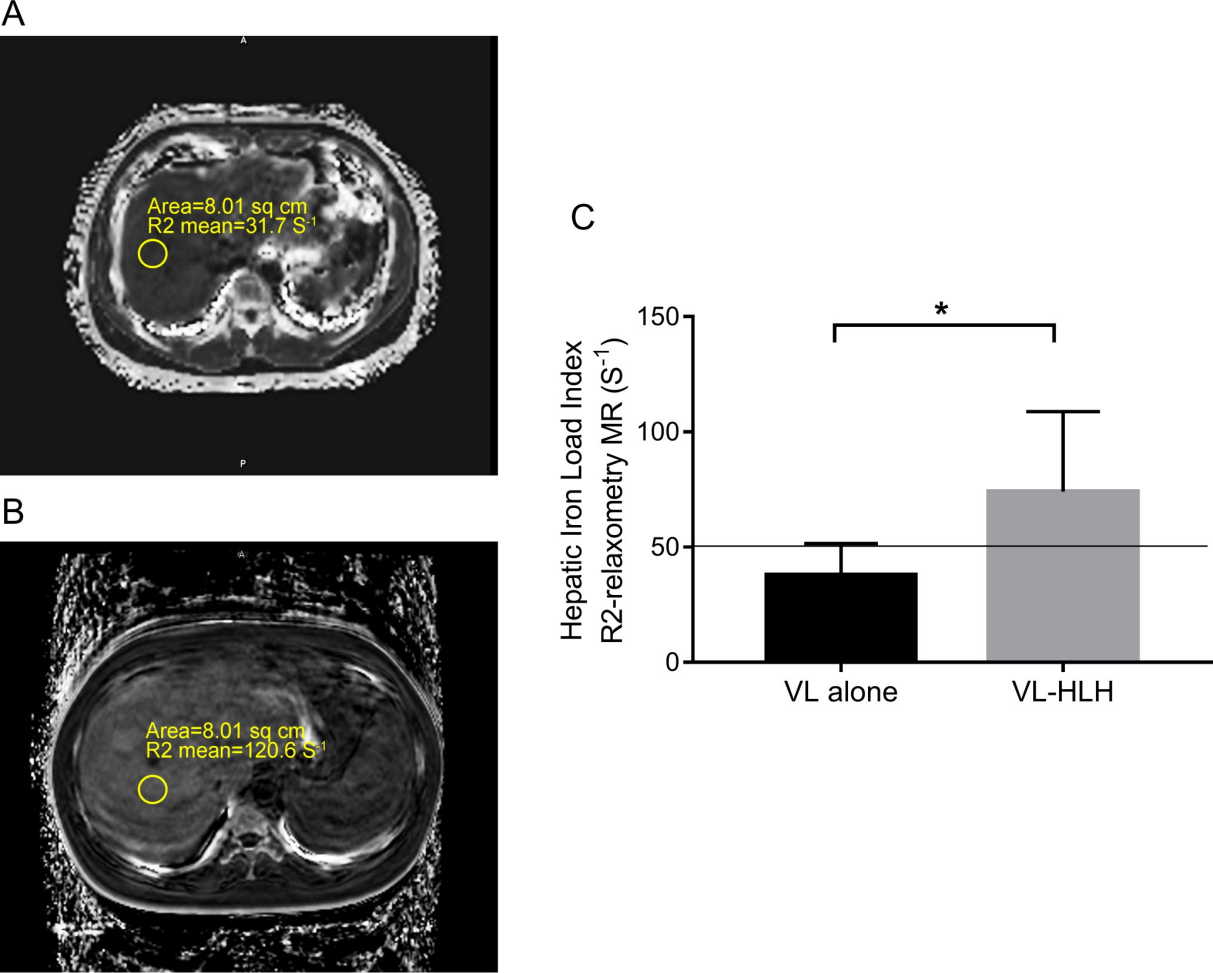
Hepatic iron load in VL alone and VL associated HLH patients with the magnetic resonance imaging. (A) Hepatic iron load for one of VL male patients was calculated by R2 relaxometry MR was 31.7 S^-1^, below the threshold of 50 S^-1^. (B) Hepatic iron load for one of VL associated HLH male patients was calculated by R2 relaxometry MR was 120.6 S^-1^, far above the threshold of 50 S^-1^. (C) Hepatic iron load statistical measurement in the VL and VL associated HLH groups (Normal range < 50 S^-1^). The chart indicated VL associated HLH patients suffered from high incidence of iron overload in the liver.

**Fig 4:**
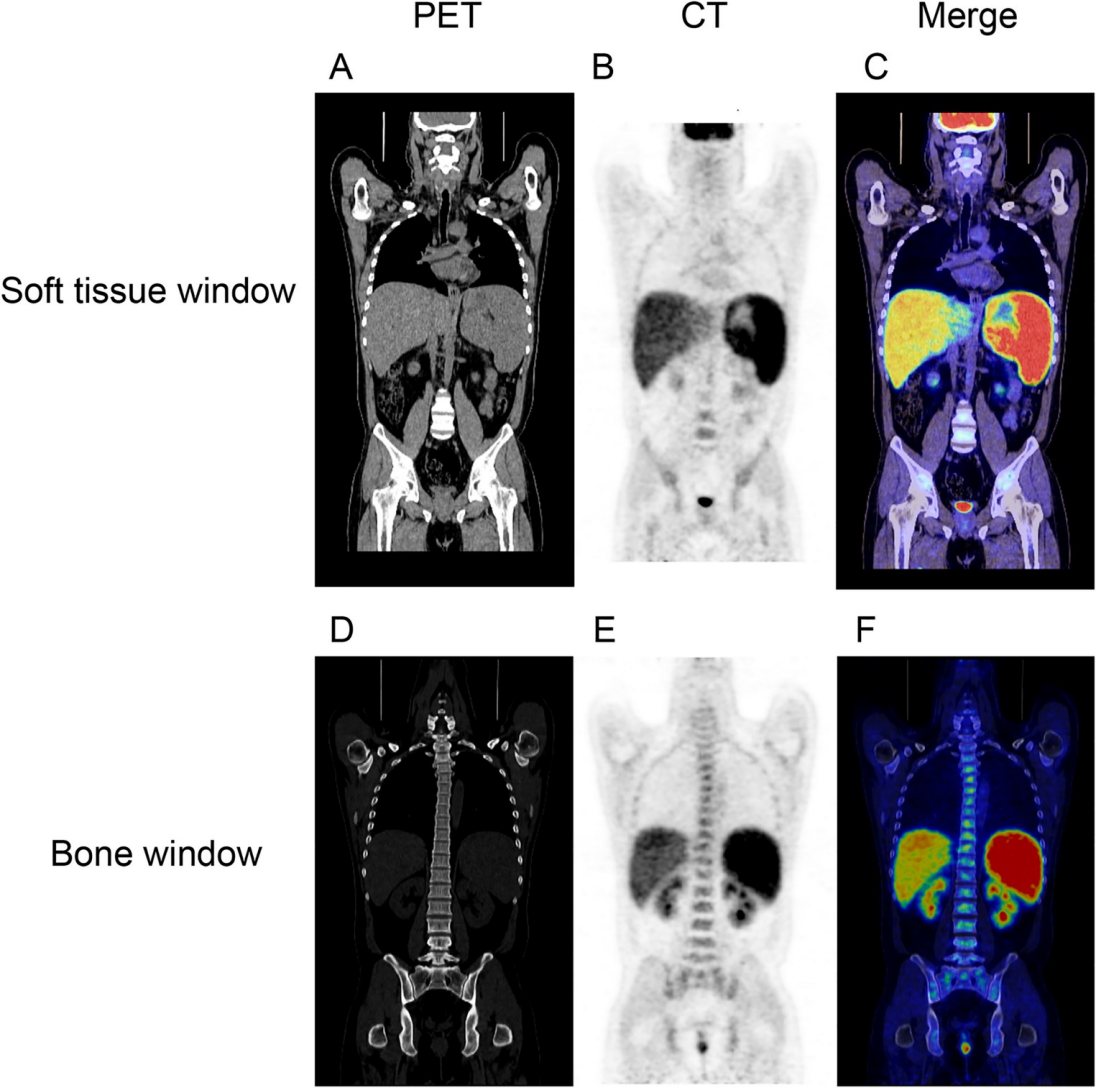
FDG PET/CT scanning in the VL associated HLH case revealed marked hepatosplenomegaly and elevated diffuse uptake in liver. (A) (SUV max = 4.3) (reference range, 2.3 to 3), in spleen (SUV max = 5.6) (reference range ≤2) and in spinal column (SUV max = 2.5) (reference range ≤1) (B, E), the merge image indicated functional disorder without organic lesion (D) in the merge image (C and F).

Both ultrasound and CT scanning revealed all VL cases had homogeneous enlargement of liver and spleen with mild hyperechoic or hyperintensity mass. There is no significant difference between VL patients with or without HLH. Interestingly, more cases with VL associated HLH (52.94%, 9/17) had hepatosplenomegaly with iron overload in the magnetic resonance imaging (MRI) than VL cases (14.81%, 4/27) with significant difference (**Fig 3**) (*P*<0.05). Moreover, **Fig 4** shows the typical image of VL associated HLH with hepatosplenomegaly (**Fig 4A**) detected by FDG-PET/CT scanning. FDG diffuse uptake in liver and spleen with the soft tissue window phrase (**Fig 4B**) and spinal column with bone window phrase (**Fig 4E**) were obviously elevated, which indicates functional disorder without organic lesion (**Fig 4D**) in the merge image (**Fig 4C and 4F**).

### Hemophagocytosis and parasitological examination

**Fig 5:**
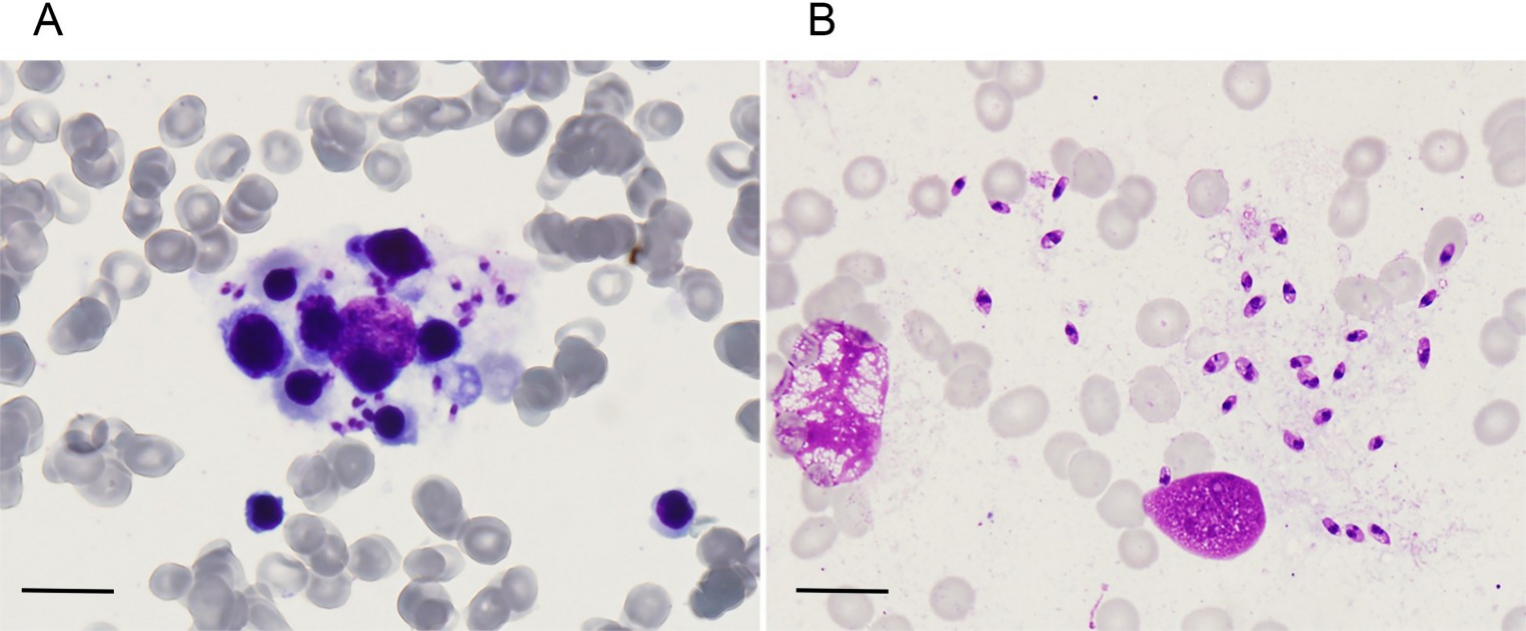
The bone marrow biopsy identified hemophagocytosis and *Leishmania* amastigotes in the VL associated HLH and VL patient. (A) *Leishmania* amastigotes was identified in bone marrow smear of VL associated HLH cases with polychromatic or orthochromatic erythroblasts and amastigotes inside under microscope. (B) Only *Leishmania* amastigotes was identified in the VL cases in the bone marrow smear (Giemsa stain, 100×). Scale bar, 10 μm.

Hemophagocytosis is the typical phenomenon in the HLH patients. In this study, 88.23% (15/17) of VL associated HLH cases revealed hemophagocytosis in the bone marrow biopsy with polychromatic or orthochromatic erythroblasts and amastigotes inside (**Fig 5A**), while VL cases only displayed *Leishmania* amastigotes in the bone marrow smear (**Fig 5B**).

Microscopy detection of amastigotes in bone marrow smear had been recognized as direct tool to determine *Leishmania* infection. In this study, we have shown that the anti-rk39 IgG detection was more sensitive compared to the traditional microscopy method in both VL and VL associated HLH groups, but without statistical significance (**Table 3**). There was not any difference between the two groups in the serological test and microscopy method. Additionally, the protozoa were identified by conventional PCR and sequencing as *L*. *infantum* in all 44 individuals (**S1 Fig**). Interestingly, both the culture positive rate in NNN medium and the *Leishmania* load quantified by qPCR displayed significant elevation in the VL associated HLH group than VL group (**Table 3**).

### Treatment and outcomes

When the *Leishmania* infection was identified, all 44 patients were intramuscularly treated with anti-*Leishmania* Sodium Stibogluconate for the consecutive 6 days as one course (10 mg/kg/day for adults and 20 mg/kg/day for children). If parasite remained in the bone marrow smear, another one or more courses of 6 days treatment were given. Patients with VL associated HLH were received methylprednisolone, cyclosporine A and etoposide in addition to the anti-parasite chemotherapy.

Finally, 39 patients including all 27 VL alone cases and 12 VL associated HLH cases were fully recovered based on the disappearance of clinical symptoms, physical sign including hepatosplenomegaly and negativity of *Leishmania* detection by microscopy and DNA amplification. Unfortunately, five patients with VL associated HLH died although the adequate comprehensive care and chemotherapy were provided (**Table 3**).

## Discussion

Hemophagocytic lymphohistiocytosis is a life-threatening syndrome characterized by pro-inflammatory cytokine secretion, hyperinflammatory and multiple organ damages. It can be caused by infection, malignancy or rheumatologic conditions except for the inherited familial factors [17]. It has been reported that leishmaniasis could cause HLH and result in fatal consequence [18]. However, it is not well characterized for the clinical and laboratory features of the visceral leishmaniasis associated HLH. Here we analyzed the clinical characterization of VL associated HLH compared with visceral leishmaniasis only, to better understand this serious complication of *Leishmania* infection [4,14,15,19]

VL associated HLH was diagnosed based on the diagnostic criteria for both HLH and leishmaniasis. The most typical findings of HLH are hemophagocytosis, fever, hepatosplenomegaly and cytopenias. Some HLH patients also show hypertriglyceridemia, coagulopathy with hypofibrinogenemia, liver dysfunction, elevated levels of ferritin and serum [15]. Since VL associated HLH has dead consequence, it is very important to get early diagnosis and timely treatment, however, it is difficult to receive definite diagnosis of VL associated HLH since the clinical symptoms and manifestations could be overlapped between HLH and VL even though we observed some characteristic features of VL associated HLH compared with VL alone in this study. We observed that the VL associated HLH patients had higher chances of bleeding (78.21%), hepatomegaly (52.94%) compared to VL alone (29.62% for bleeding and 22.22% for hepatomegaly) with significant difference. Besides, we identified triglycerides (TG) and low-density lipoprotein (LDL-C) were significantly elevated in the VL associated HLH cases than those in VL patients, indicating the lipid metabolism was seriously harmed in patients with VL associated HLH. Ferritin and LDH in sera reflected the inflammatory level and tissue damage in the infectious diseases. In this study, the ferritin and LDH levels were elevated in both groups, but significant higher in VL associated HLH cases than in VL cases, which indicated that hypertriglyceridemia and hyperferritinemia are considered as important parameters in the determination of HLH and for the differentiation between VL associated HLH and VL alone [15]. Hemophagocytosis is the typical phenomenon of HLH, but is not absolutely needed for the diagnosis of HLH. Two of 17 VL associated HLH cases did not show hemophagocytosis but met other criteria of HLH diagnosis. If patient from endemic area is definitely diagnosed with *Leishmania* infection (parasite identification), and occurs with 5 of other criteria for HLH diagnosis [15], the VL associated HLH should be considered and the anti-parasite treatment as well as HLH treatment should be initiated sooner the better to reduce the mortality.

Cytokines determinations seem to reveal more significant difference between VL associated HLH and VL only which may assist their differentiation. In this study, we found that patients with VL associated HLH exhibited excessive immune responses characterized by the much higher serological levels of immunoglobulin G and Th1 cytokines (TNF-α, IFN-γ, IL-1beta, IL-6, IL-8, IL-12p70), Th2 cytokines (IL-4) and Th17 cytokine (IL-17, IL-23) compared to VL without HLH (**Fig 2**). The results indicate that HLH may occur when NK cells, macrophages or T cells are consistently stimulated by the infection and resulting in hypercytokinemia and immune-mediated injury of multiple organ systems [6]. Although the precise mechanisms still remain unknown, it seems that the Th1/Th2 imbalance and the cytokines storm produced by the abnormal T-lymphocyte response induced by the parasite may result in uncontrolled activation of the mononuclear phagocyte system leading to HLH. The inflammatory cytokines such as interferon-γ, IL-1beta, IL-6, IL-8, and TNF-α are involved with leishmaniasis severity [20]. IL-17 and IL-23 expression is considered as a marker of Th17 response, Th17 cells are a crucial modulator of adaptive immunity against *Leishmania* parasites to recruit neutrophil to the infection site and to play a dual role at the site of infection [21].Therefor, our results indicate that not only Th1/Th2, but also Th17 are involved in the protective immunity against *Leishmania* infection, which is possibly involved in the immunopathology and tissue damage in the *Leishmania*-triggered HLH.

The image pattern of VL associated HLH cases has not been well previously described except for the hepatosplenomegaly. Previous VL case report showed multiple nodules with concentric rings in the spleen on T2-weighted imaging (T2WI), with no obvious diffusion restriction on diffusion weighted imaging (DWI) [22]. In this study, we found the high incidence of diffuse hepatosplenomegaly with iron overload detected by the MRI scanning in VL associated HLH cases. Iron overload have been recognized as the hallmark of severe infections such as protozoa and helminths [23,24]. It is also associated with hyperferritinemia of HLH [25]. Ferritin is a high molecular weight serum protein that contains iron commonly measured to assess tissue iron storage. It is also a positive acute phase reactant to reflect inflammatory conditions. The extreme elevations of serum ferritin may result from the activation of inflammatory cytokines that mediate the acute phase response, but in turn to mediate the pro-inflammatory process by inducing the expression of pro-inflammatory cytokines [26]. Thus, iron overload with hepatosplenomegaly detected in this study may reflect the severe conditions in the *Leishmania* infection and its associated HLH. Several studies have demonstrated the pattern of Fluorine-18 Fluorodeoxyglucose Positron Emission Tomography/Computed Tomography (F18 FDG-PET/CT) in the visceral leishmaniasis [27–29]. Diffuse splenic and bone marrow uptake of FDG is the main feature in the VL patients, and VL associated HLH cases presented more significantly elevated diffuse uptake in the liver, spleen and spinal column. This special PET/CT scanning, for what we believe is the first time, describes the important features of the VL associated HLH cases.

The identification of *Leishmania* parasite is the key to diagnose *Leishmania* associated HLH. The typical diagnostic method for *Leishmania* infection is the microscopy with identification of amastigotes in direct bone marrow smears or promastigotes found *in vitro* culturing in Nicole-Novy-McNeal medium at 23 to 25°C [30]. However, the identification of parasite under microscope is time-consuming and needs experienced technician, usually with low identification rate. Recently developed molecular detections such as conventional PCR and qPCR are recommended with increased sensitivity and specificity [14,31]. In this study, we found there was no difference in the detection efficiency of *Leishmania* between VL alone and VL associated HLH groups using immunological and microscopy method. However, we found that positive rate of parasite culture and quantification by qPCR significantly were increased in the VL associated HLH cases, indicating the higher infection density contributes to the onset of *Leishmania* associated HLH. Moreover, the results suggest that quantitative detection of *Leishmania* DNA in bone marrow samples using qPCR may assist the diagnosis of *Leishmania* associated HLH and should be included in HLH diagnostic protocols.

The treatment principles for VL associated HLH patients is to remove parasite by using anti-parasite therapy combined with immunosuppression treatment to suppress the life-threatening inflammatory process that underlies HLH. Thus, anti-Leishmanial treatment should be initiated as soon as the *Leishmania* parasite is identified in patients with HLH conditions. Sodium Stibogluconate (SSG) is still the first line therapeutic drug to treat leishmaniosis in China [13], although the amphotericin B or liposomal amphotericin B is recommended as well. Six-day course therapy with SSG was proved to be effective and safe in the treatment of VL [13]. Besides the anti-*Leishmania* treatment, anti-inflammatory therapy to reduce cytokine storm and excessive immune responses is necessary. Chemotherapy including methylprednisolone, cyclosporine A and etoposide is recommended particularly due to corticosteroids as the important components [15,32,33]. Indeed, the combination of anti-*Leishmania* and anti- inflammatory therapy facilitated the remission in the VL associated HLH patients [34].

This study was performed in a selected population of 44 VL patients with or without HLH that may limit the generalization of the findings. However, due to the rarity of the VL and insufficient information of VL triggered HLH available so far, we hope this study offers insights into clinical and laboratory characteristics of secondary HLH induced by *L*. *infantum* infection.

In conclusion, VL associated HLH is a rare but potentially fatal condition. The parasite culturing and the qPCR detection of DNA from bone marrow samples of HLH patients are critical for the diagnosis of VL associated HLH. Prompt treatment with anti-*Leishmania* agents and chemotherapy could improve the prognosis of patients with VL associated HLH.

## Supporting information

S1 Fig Gel electrophoresis of PCR products for *Leishmania* mkDNA region from bone marrow of each patient. (A) 17 samples form VL associated HLH patients were amplified and identified as *Leishmania* infection (B) 27 samples form VL alone patients were amplified and identified as *Leishmania* infection. The PCR products in two groups were then sequencing as *L*. *infantum*. (Distilled water as negative control and *L*. *infantum* as a positive control).
(TIF)

S1 Table Original data for Table 2, Table 3, Fig 2, Fig 3 and Fig 4. (XLSX)
