# Supplementary figures and images for: Clinical and laboratory characteristics of hemophagocytic lymphohistiocytosis induced by Leishmania infantum infection
Source: PLoS Negl Trop Dis. 2021 Nov 4;15(11):e0009944. doi: 10.1371/journal.pntd.0009944 (PMC8594843; doi:10.1371/journal.pntd.0009944)

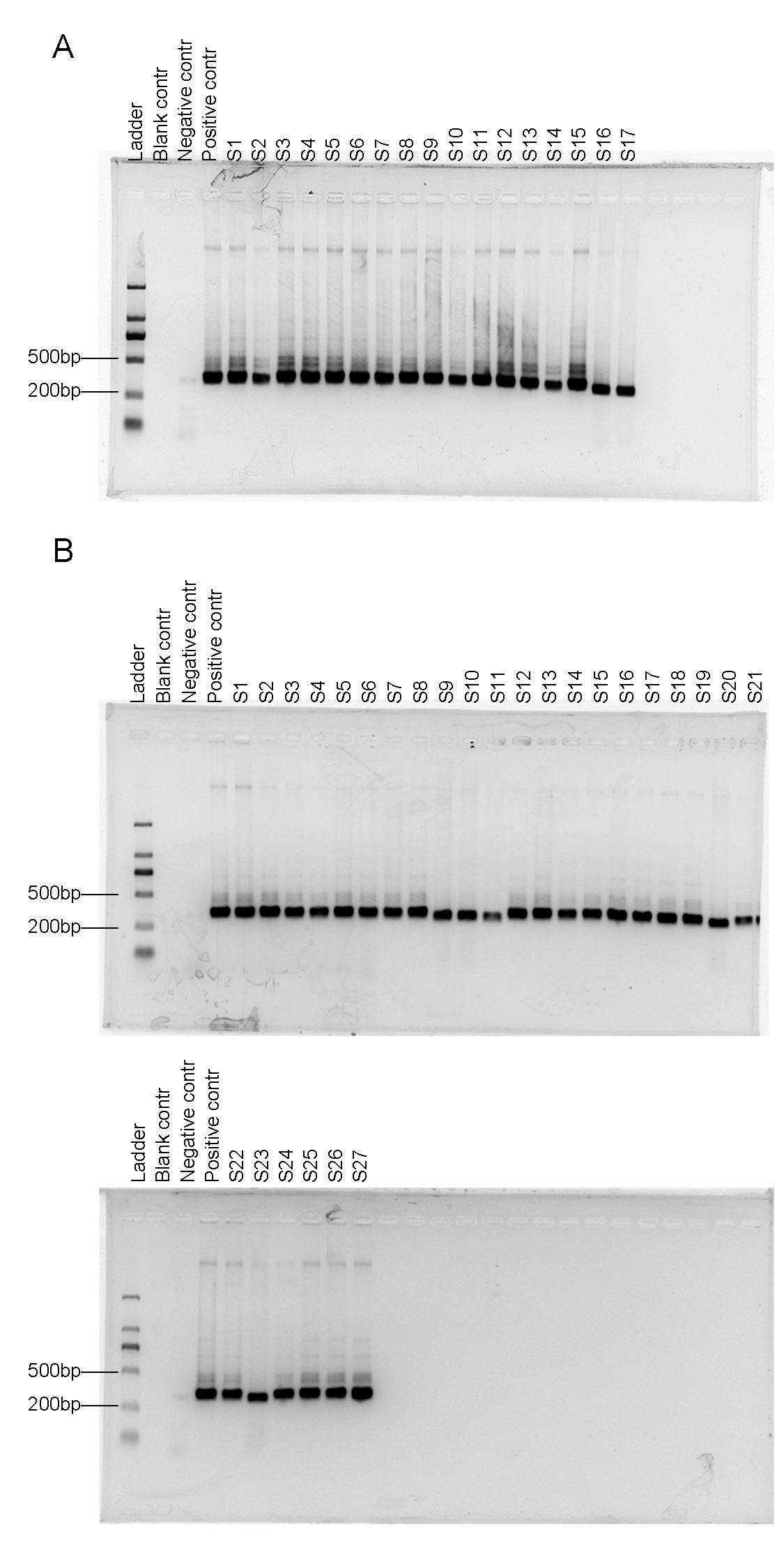

Supplement: S1 Fig — (A) 17 samples form VL associated HLH patients were amplified and identified as Leishmania infection (B) 27 samples form VL alone patients were amplified and identified as Leishmania infection. The PCR products in two groups were then sequencing as L. infantum. (Distilled water as negative control and L. infantum as a positive control). (TIF) [file pntd.0009944.s001.tif]
